# Closing the Gap Between Theory and Practice: Conceptualisation of a School-Based Intervention to Improve the School Participation of Primary School Students on the Autism Spectrum and Their Typically Developing Peers

**DOI:** 10.1007/s10803-021-05362-5

**Published:** 2021-12-04

**Authors:** Amy Hodges, Reinie Cordier, Annette Joosten, Helen Bourke-Taylor

**Affiliations:** 1 Curtin School of Allied Health, Curtin University, Perth, WA Australia; 2 Department of Social Work, Education and Community Wellbeing, Northumbria University, Newcastle upon Tyne, UK; 3 School of Allied Health, Australian Catholic University, Melbourne, VIC Australia; 4 Department of Occupational Therapy, School of Primary and Allied Health Care, Monash University, Frankston, VIC Australia

**Keywords:** Psychosocial intervention, Schools, Autism, Theoretical model, Intervention development

## Abstract

Limited interventions exist that support student’s school participation. This paper describes a theoretical model of school participation and the iterative process that led to the development of an intervention that aims to improve the school participation of students on the autism spectrum and their typically developing peers. Literature on autism, school participation and intervention research were integrated to develop a theoretical model. Focus groups, a Delphi study, online surveys, and reference group consultation helped to develop and refine the intervention*.* A novel school-based intervention was developed. The impetus to develop interventions with a strong theoretical rationale is discussed.

**Supplementary Information:**

The online version contains supplementary material available at 10.1007/s10803-021-05362-5.

## Introduction

School participation is essential to students’ social, emotional and academic development (Frederickson et al., 2007). In recent years there has been growing concern about the school experiences of students on the autism spectrum. This research indicates that students on the autism spectrum experience significant school participation restrictions and are more likely to experience bullying, less social support and more frequent suspensions compared with typically developing peers (Humphrey & Symes, 2010; Jones & Frederickson, 2010). Persistent challenges participating at school can lead to students feeling like they do not belong at school, which can have a significant long-term impact on student outcomes (Shochet et al., 2006). However, there are limited interventions available that specifically aim to increase student’s participation at school (Centers for Disease Control & Prevention, 2009).

The development of interventions that aim to improve students’ school participation requires an understanding of the construct of school participation and factors that support or hinder students’ experiences. This is critical, as without a clear understanding of the construct, we cannot be sure interventions are targeted appropriately. In this paper, we present a theoretical model that illustrates the interaction between characteristics of autism and factors that promote school participation. We then describe how we used this theoretical model to engage in a multi-stage iterative process to develop a school-based intervention aiming to improve the school participation of primary school students on the autism spectrum and their typically developing peers.

## The Research Team

The development of the theoretical model and resulting intervention was led by authors of this paper. The primary author is a registered occupational therapist with clinical experience working with children and young people with a range of disabilities, specialising in providing community based consultative services to support school aged students on the autism spectrum, their families, and educators. Professor Reinie Cordier’s research focuses on promoting the social inclusion of children with various developmental disabilities, such as autism, measurement and psychometrics and developing evidence-based psychosocial interventions. Associate Professor Annette Joosten has extensive clinical and research experience in area of autism, early intervention, and the impact autism has on participation. Associate Professor Helen Bourke-Taylor has research experience in school participation and the involvement of children with atypical learning needs. The expertise of the research team is important to describe as it provides context and validates the theoretical model and intervention as an expert led, research informed initiative.

## The Proposed Theoretical Model of School Participation and Autism

The theoretical model of school participation and autism (MSPA) was constructed following a critical appraisal of the literature relating to autism, school participation and intervention research. Authors reviewed all studies included in a systematic literature review of the psychometric properties of school connectedness measures (see review for search terms and studies included; Hodges et al., 2018). Additional searches were conducted using a range of databases such as CINAHL, Embase, ERIC, Medline, PsycINFO, to identify studies exploring the relationship between characteristics of autism and school participation, as well as intervention techniques used and found to be effective in facilitating the school participation of students on the autism spectrum. All studies were independently reviewed by the primary author, and then by the research team, based on a pre-set criteria to determine the strength of the relationship between factors illustrated in the MSPA. Relationships in the MSPA were considered ‘strong’ if more than 70% of studies reviewed showed a direct relationship between factors in the MSPA (e.g., the social communication skills of students on the autism spectrum improved following a peer mediated intervention), the purpose of the study was clearly linked to factors in the MSPA, the quality of studies was quasi-experimental or higher, and there were autism specific findings. Relationships were considered ‘emerging’ if less than 70% of studies reviewed showed a direct relationship, the purpose of the study was not clearly linked to factors in the MSPA, the quality of studies was lower than quasi-experimental or only used qualitative methodology, and findings were not autism specific. Integrating literature on autism, with literature on school participation and intervention research enabled us to construct an evidence-based theoretical model that depicts the interactive process between characteristics of autism and factors that promote school participation.

The MSPA is based on Imms and colleagues’ (2016) framework of participation, called the family of Participation and Related Constructs (fPRC), which was developed following a systematic literature review of language, definitions and constructs used in participation intervention research with children with disabilities (Imms et al., 2015). The MSPA extends the fPRC by applying the fPRC to students on the autism spectrum in the school environment. According to the fPRC, participation comprises two essential components: “*attendance*—defined as ‘being there’ and measured as frequency of attending, and/or the range or diversity of activities; and *involvement*—the experience of participation while attending” (Imms et al., 2016, p. 18). In the context of education, this means being actively engaged in activities, tasks and routines that are typical for students of that age in a given education system, as well as a subjective feeling of belonging, and being active in the school environment (Libbey, 2004). Merely being present in a mainstream classroom does not lead to participation and is not indicative of successful inclusion (Symes & Humphrey, 2012).

Based on the fPRC, several intrinsic factors can influence and, in turn, are influenced by participation (Imms et al., 2016). Intrinsic student factors impacting school participation include students’—*activity competence* (i.e., the ability to execute an activity to an expected standard; Imms et al., 2016), *sense of self* (i.e., personal perceptions related to students confidence, satisfaction, self-esteem and self-determination; Imms et al., 2016) and *preferences* (i.e., interests or activities that hold meaning or are of value; Imms et al., 2016). These factors are considered antecedents to, and consequences of, school participation—they influence future participation and are influenced by past and present participation (Imms et al., 2016). For example, to participate in an activity at school students must have a degree of interest; however, through participation students’ interest may increase or they may develop new interests that hold meaning or are of value to them.

In addition to extending the fPRC to schools, the MSPA includes students’ sense of school connectedness as an *additional intrinsic student factor,* based on a large body of literature emphasising the significant impact reduced school connectedness has on students’ school participation and student outcomes (Furlong et al., 2003; Maddox & Prinz, 2003; Shochet et al., 2006). The MSPA also acknowledges that all participation occurs within a contextualised setting and recognises the moderating and mediating impacts students’ school, family, and community environments have on students’ school participation (Anaby et al., 2014; Colver et al., 2012; Eriksson, 2005). Environmental factors, such as the impact unexpected changes in the curriculum, attendance at school events such as sports carnivals, and the implementation of evidence based intervention techniques has on students school participation, have been explicitly illustrated in the MSPA. Broader social and cultural environmental factors, however, such as peer and teacher understanding, awareness and acceptance of autism, and teachers knowledge, attitudes and skills in supporting students with diverse learning needs, have not been explicitly illustrated in the model due to layout restrictions, but are recognised as factors that can impact student school participation.

**Fig. 1:**
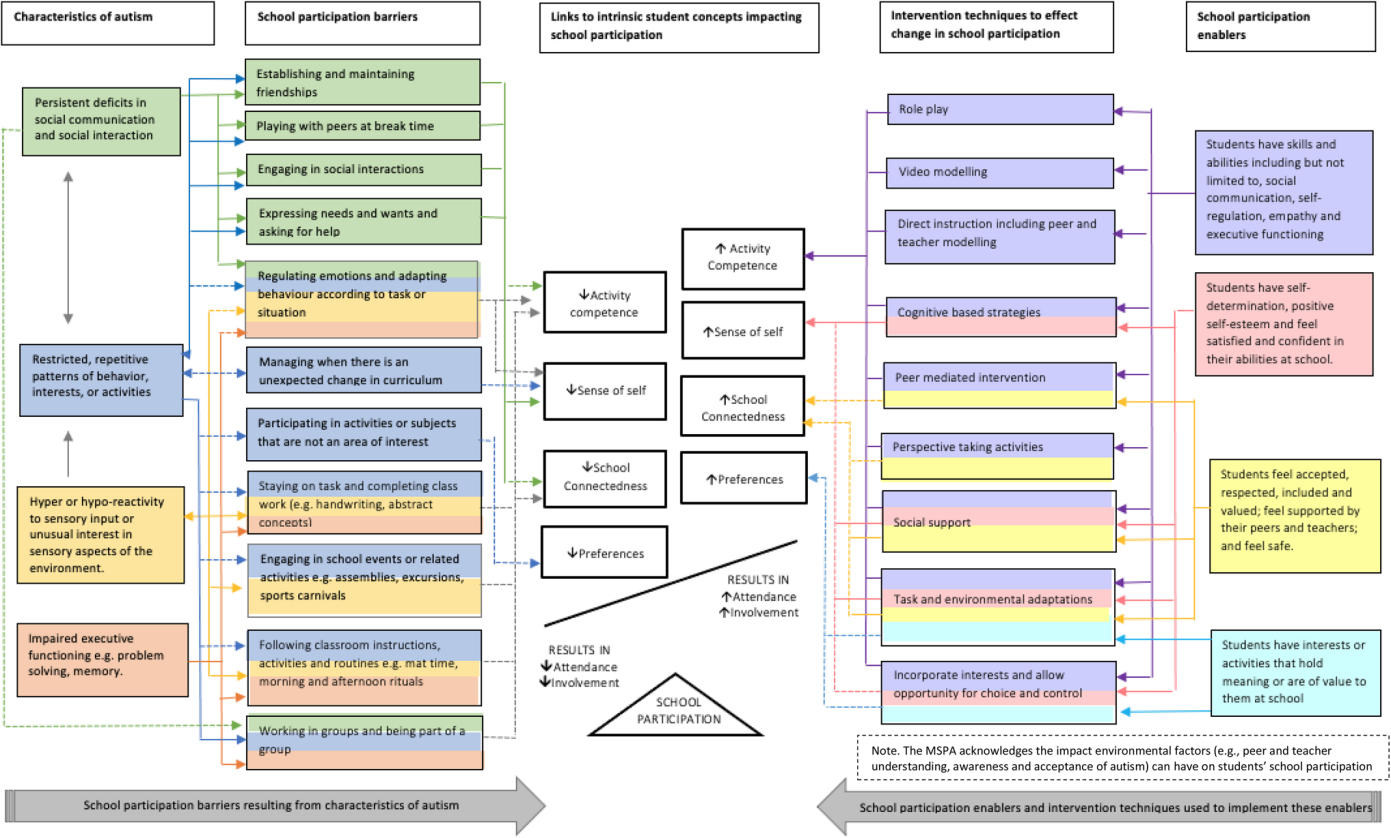
The proposed model of school participation and autism (MSPA)

Figure 1 outlines the MSPA. The centre of the model represents the school participation transaction and shows that any reduction in intrinsic student factors (i.e., due to characteristics of autism or environmental factors) needs to be offset by school participation enablers (i.e., intervention techniques). Uni- and bi-directional arrows are used to illustrate relationships between factors and a colour coding system has been used to assist with readability and interpretation. Solid lines between factors indicate that the relationship between factors is strongly supported in the literature, whereas dotted lines indicate the relationship between factors is still emerging in the literature.

School participation barriers that result from characteristics of autism (illustrated from left to centre in Fig. 1), such as difficulty establishing and maintaining friendships, are specifically linked to intrinsic student factors in the centre of the model. For example, literature suggests difficulty regulating emotions impacts student’s capacity to learn effectively (Laurent & Rubin, 2004) and impacts the development of social, communication and problem-solving skills (i.e., activity competence; Prizant & Wetherby, 2005). This relationship received a dotted line as relationships identified in the literature were indirect or inferred and not always autism specific.

School participation enablers and intervention techniques used to implement these enablers (illustrated from right to centre in Fig. 1), are also linked to intrinsic student factors as depicted in the centre of the model. For example, literature suggests peer mediation is a robust method for teaching and improving academic and social communication skills, as well as improving peer acceptance and reducing social isolation (Bene et al., 2014; Wang et al., 2011). The relationship between peer mediated intervention and activity competence received a solid line as there have been several autism-specific experimental studies conducted outlining strong direct relationships as well as reviews and meta-analyses (Bambara et al., 2016; Banda, Hart, & Liu-Gitz, 2010; Bene et al., 2014; Rodriguez-Medina et al., 2016; Strain et al., 1979; Wang et al., 2011). Conversely, the relationship between peer mediated intervention and school connectedness received a dotted line as relationships in the literature were largely inferred and the purpose of studies were not clearly linked to the concept of school connectedness (Kasari et al., 2012; Rodriguez-Medina et al., 2016). Inconsistency in the way school connectedness is conceptualised and defined, however, may contribute to lack of strong evidence to support this relationship. To effect change in student school participation, the MSPA proposes school participation enablers as implemented through intervention techniques need to offset the barriers that result from characteristics of autism. The proposed model is described briefly below in relation to four intrinsic student factors of school participation and autism.

### Activity Competence and Autism

The school environment is complex and requires many skills to successfully navigate. Autism can impact the development and performance of several skills, such as social communication, which can significantly impact students’ ability to participate at school (Saggers et al., 2011, 2016). Social communication participation restrictions can include difficulty establishing and maintaining friendships at school, engaging in social interactions, expressing needs and wants and asking for help at school (Hodges et al., 2020). Literature suggests students on the autism spectrum are less likely to initiate social interactions and spend a larger proportion of time engaging in non-social play at school (Koegel et al., 2012). Students’ school participation can be further impacted by hyper or hypo reactivity to sensory input with noise, touch, and the ability to stay still, identified as sensory preferences, significantly impacting students’ learning and performance at school (Saggers et al., 2016). Furthermore, impaired executive functioning skills, such as problem solving and attention, can result in students having difficulty adapting their behaviour, following instructions, and being part of a group (Torrado et al., 2017; Zingerevich & LaVesser, 2009).

Several effective intervention techniques have been identified to improve the social communication, play and problem-solving skills of students on the autism spectrum including peer mediation (e.g., large effect size (ES) = 1.3, 95% CI; Wang et al., 2011); role play (e.g., medium ES = 0.92, 95% CI; McCoy et al., 2016), video modelling (e.g., large ES = 1.22, 95% CI; Wang et al., 2011), and direct instruction (Ganz & Flores, 2009; Klinger, Klinger, & Pohlig, 2007). Peer mediated interventions facilitate active student engagement by providing students with frequent opportunities to respond, and provide prompts and feedback (Bene et al., 2014; Wang et al., 2011). Results from a meta-analyses found peer mediated instructional arrangements to have a significant impact on students on the autism spectrum in academic content areas (e.g., reading, comprehension), as well as social communication skills and reducing problem behaviours with an average ES of 0.82 of all studies reviewed (95% CI; Bene et al., 2014).

### Sense of Self and Autism

While skills are necessary to be able to participate at school, another key factor impacting student school participation is students’ sense of self, including students’ confidence (i.e., students’ perceived competency, skill and capability to deal effectively with various situations; Shrauger & Schohn, 1995), satisfaction (i.e., short term attitude resulting from an evaluation of students educational experience, services and facilities; Weerasinghe et al., 2017), self-esteem (i.e., overall subjective sense of personal worth or value; Blascovich & Tomaka, 1991) and self-determination (i.e., ability to think and make decisions without external influences; Hui & Tsang, 2012; Imms et al., 2016). Lack of structure and predictability in the school environment, students’ awareness of limited social relationships and difficulties connecting with peers, and persistent challenges participating at school can result in students feeling less satisfied and confident at school which can lead to a negative sense of self (Humphrey & Lewis, 2008). As a result of these challenges, students on the autism spectrum are more likely to experience bullying and social isolation (Rowley et al., 2012), leading to increased risk of anxiety and depressive symptomatology (Shochet et al., 2006).

Interventions utilising a strengths-based approach that aim to increase students’ self-awareness of differences and provide opportunities for students to make choices, in line with principals of social and emotional learning (Jones & Bouffard, 2012; Pasi, 2001; Romasz et al., 2004), have been found to contribute to an improved sense of self for students on the autism spectrum (Niemiec & Ryan, 2009; Reutebuch et al., 2015). Cognitive based strategies such as seeking evidence for and against the validity of thoughts, identifying consequences for holding a particular belief, and categorising thought distortions have strong evidence to support their effectiveness in improving self-esteem, reducing anxiety symptoms, self-report school anxiety and social worry for students on the autism spectrum (Chalfant et al., 2007; Lee et al., 2016; Luxford et al., 2016; Wood et al., 2009). For example, a study by Wood et al., (2009) reported a significant reduction in anxiety symptoms for students on the autism spectrum following a cognitive behavioural therapy intervention with a large reported ES of 2.46 (Cohen, 1988). Finally, task and environmental modifications such as the use of multi-media to increase student enjoyment (Hiemann et al., 1995) and providing access to a range of activities that cater to students diverse interests, in line with principals of universal design (Center for Applied Special Technology, 1998; Orkwis, 2003; Spooner et al., 2007), have also been found to increase students sense of self (Eime et al., 2013; Hinchliffe et al., 2016; Mahoney et al., 2003).

### School Connectedness and Autism

The extent to which students feel valued and cared for in their school community, referred to as school connectedness, is considered a predictor as well as an outcome of student school participation (Ciani et al., 2010). A study by Wainscot and colleagues (2008) reported 90% of students on the autism spectrum felt they were disliked by someone at school. Studies also report students on the autism spectrum have fewer friends and that their friendships are of poorer quality (Kasari et al., 2011).

Modification to the social and physical environment, such as improving peer and teacher awareness and understanding of autism, has been linked to improved sense of connectedness at school (Batten et al., 2006). Peer mediated interventions focusing on increasing peer acceptance of autism and basic strategies to promote inclusion have also been found to improve the school connectedness of students on the autism spectrum (Harper et al., 2008; Owen-DeSchryver et al., 2008). For example, a study by Kasari and colleagues (2011) reported students on the autism spectrum received more friend nominations from their peers and were observed to be less isolated in the playground following the implementation of a peer mediated intervention.

### Preferences and Autism

The motivation to participate rests on the premise that there are interests or activities at school that hold meaning or are of value to students (Imms et al., 2016). Students on the autism spectrum often have intense interests and a preference for sameness, which can impact their ability to participate in activities or subjects that are not an area of interest and manage when there is an unexpected change at school (Koegel et al., 2010). These challenges often result in students engaging in behaviours that can be disruptive in the school environment, which further impacts students’ capacity to participate at school (Saggers et al., 2016). Furthermore, the school environment is often highly structured with limited flexibility in how the curriculum is taught; limiting students’ capacity to make choices and feel in control. Incorporating students’ interests and allowing choice and control in interventions has been found to improve students’ motivation, task completion and socialisation and reduce disruptive behaviour (Koegel et al., 2013; Reutebuch et al., 2015; Ulke-Kurkcuoglu & Kircaali-Iftar, 2010).

Current interventions for students on the autism spectrum tend to focus on targeting students’ skills in isolation, with an expectation there will be a flow-on effect on students’ participation (social skills; Mackay et al., 2007; McConnell, 2002; Ostmeyer & Scarpa, 2012). The MSPA highlights that to effect change in students’ school participation, a holistic approach using evidence-based intervention techniques is required, targeting not only students’ skills (i.e., activity competence), but also psychological aspects (i.e., sense of self, school connectedness and preferences) of students’ school experiences. We used the MSPA as a theoretical foundation to guide the development of a school-based intervention aiming to improve school participation of primary school students on the autism spectrum and their typically developing peers from conceptualisation to implementation in the school environment.

## The Multi-stage Iterative Process of Developing the School-Based Intervention

A series of research activities and studies informed the development of the school-based intervention, which involved: (a) a literature review of effective components of existing school-based interventions; (b) regular consultations with a consumer and stakeholder reference group (CSRG); (c) focus groups with parents and educators to explore their perspectives on the school participation of students on the spectrum and gain general recommendations regarding the intervention (Hodges et al., 2020); (d) a national 2-round Delphi study to gain consensus on the application of the fPRC to students on the autism spectrum and recommendations on the content, delivery and feasibility of the intervention (Hodges et al., 2021); and (e) feedback from students, parents, educators on intervention resources.

**Fig. 2:**
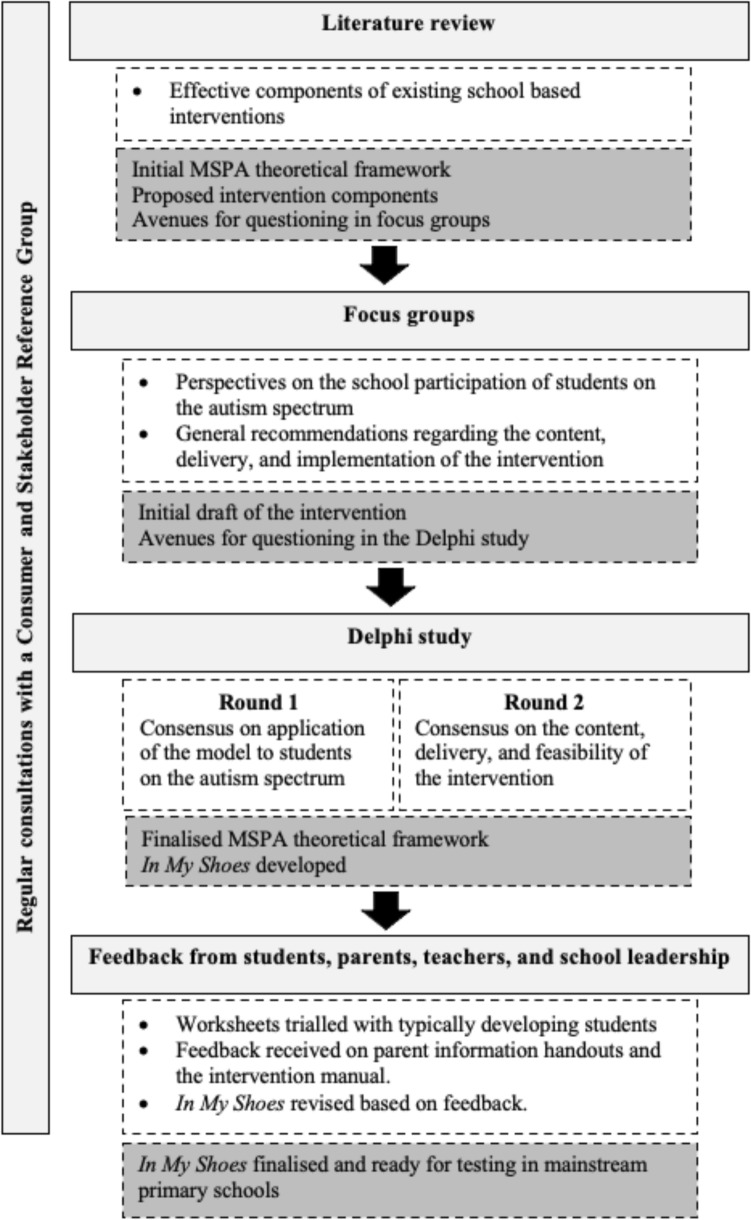
Multi-stage iterative process of intervention development

Ethics approval was obtained from the Human Research Ethics Committee at Curtin University (HREC2016-0150) and permission granted from relevant schooling sectors, such as Catholic Education Western Australia and the Association of Independent Schools Western Australia (AISWA) prior to data collection. Figure 2 illustrates the multi-stage iterative process of developing the intervention and outcomes of each stage of the research, described below.

## Literature Review

### Effective Components of Existing School-Based Interventions

Research indicates school-based interventions that yield the most successful results are those that are embedded across the whole school, using a multi-modal approach (Clark et al., 2019; Goldberg et al., 2019). This approach typically involves coordinated action between “…curriculum, teaching and learning, the school ethos and environment and family partnerships” (Goldberg et al., 2019, p. 771). The primary author conducted a series of electronic database searches to identify intervention studies or reviews that reported on the effectiveness of school based interventions. Included studies were published in the last 15 years, reported on the effectiveness of intervention components of *school* based interventions but were not necessarily specific to students on the autism spectrum. Studies were independently reviewed and summarised by the primary author, and then discussed with the research team, until agreement was reached to identify core components of the intervention. These included: (a) professional learning for teachers and school leadership staff; (b) teacher-led whole class lesson plans; (c) peer training for selected peers; (d) activity ideas to incorporate key messages across the whole school; and (e) weekly parent information handouts and invitations for parents to participate in the intervention.

The provision of professional learning is imperative to support the integration and sustainability of school-based interventions (Clark et al., 2019). Teachers often report a lack of training in relation to students on the autism spectrum. For example, in a recent study in Sweden, only 14% of staff reported receiving any formal training in teaching students with neurodevelopmental disabilities (Bartonek et al., 2018). As a result, teachers often felt ill-equipped to meet student needs and deliver school-based supports. The professional learning component of the intervention includes training and ongoing support, including information related to autism, as well as specific instructions on how to implement the intervention (Hodges et al., 2020), for teachers and school leadership staff delivering the intervention.

Teacher-led whole class lesson plans were developed to immerse *all* students in learning that aims to improve students’ interpersonal empathy and ability to display behaviours that help others participate and feel included at school. School participation barriers identified in the MSPA were grouped into themes, which then formed proposed lesson topics using a strengths-based approach. For example, staying on task, completing worksheets, and following classroom instructions and routines were grouped into a theme called ‘helping each other in the classroom’. This lesson aims to support students to take the perspective of others who may find learning in class more difficult, due to difficulties with skills such as attention, self-regulation, executive functioning, and social communication. It aims to teach students how to recognise when a peer is having difficulty in the classroom and practise ways to help and learn ways to ask for help themselves when they needed it in class. Intervention techniques deemed effective for students on the spectrum such as peer mediation (Chan et al., 2009), video modelling and role play (Thompson, 2014) were incorporated into lesson plans. For example, role play was incorporated into the ‘helping each other in the classroom’ lesson, which involved an activity in this lesson requiring students to take the perspective of students who have limited verbal communication by trying to communicate what is written on a piece of paper to a partner without using any words.

Peer involvement in interventions play a critical role in promoting social interactions and friendships and creating communities where all students help each other learn (National Association of Special Education Teachers, 2020). Peer involvement also allows interventions to be delivered within a child’s natural environment; providing ongoing opportunities for students to practice their social skills and increase the likelihood skills will be generalised across settings (Chan et al., 2009; Watkins et al., 2015). While the whole class component of the intervention aims to teach *all* students to be natural peer mentors, the peer training component involves selecting a small number of peers with strong interpersonal skills to receive additional teacher-led training prior to the commencement of the intervention, to support them to provide additional support to target students in the classroom and playground.

Involving parents in school-based interventions reinforces complementary roles of families and educators and extends opportunities for learning across contexts where students spend most of their time (Goldberg et al., 2019). The parent component of the intervention involves weekly information handouts and inviting parents to participate in intervention-specific activities. At a school level, literature recommends reinforcing core concepts through non-curriculum-based activities in the school designed to promote a positive school climate (Minniss & Stewart, 2009; Rowe et al., 2007). The whole school component of the intervention includes information for school leadership staff about the importance of school involvement for student outcomes (Carrington et al., 2020; Goldberg et al., 2019) and activity ideas to incorporate key messages across the school. Prior to further development, information was obtained from students, parents, educators, researchers and clinicians via a reference group, focus groups (Hodges et al., 2020), Delphi study (Hodges et al., 2021) and online feedback surveys to develop and further refine the intervention until it was ready to test in mainstream primary schools.

### Regular Consultation with a Consumer and Stakeholder Reference Group

Throughout the intervention development process, a CSRG were consulted, which included an occupational therapist, speech therapist, teacher, deputy principal and two parents of primary school students on the autism spectrum. One parent, who had two primary school-aged children on the autism spectrum, also had a diagnosis of autism herself and had a professional background in teaching. In the beginning, the primary author met with the group to ask more general questions relating to research design and the readability of participant information sheets. As the research progressed, the primary author met with individual members of the reference group as required. For example, the deputy principal was consulted on ways to maximise school uptake of the intervention, whereas parents were consulted on their preferred use of language in the autism specific lesson plan and strategies to maximise parent engagement. The utilisation of a CSRG helped to understand consumers’ and stakeholders’ lived experiences with research and school-based supports, which helped to identify perceived barriers in implementing the intervention as well as problem-solve ways to maximise uptake of the intervention and ensuing research (Mathie et al., 2014).

Primary school students with and without autism were also involved in co-designing and co-producing intervention resources. For example, the school experiences of real-life students on the autism spectrum were explored and documented in an edited documentary style video developed in collaboration with the (name of organisation removed for peer review). Typically developing primary school aged students were also involved in intervention development, acting in a series of interactive video resources for use in the whole-class component of the intervention. Involving students in developing intervention resources was integral in ensuring the authentic lived experiences of school aged students were addressed, and that resources were relevant and suitable to end users (Consumer & Community Health Research Network, 2017).

### Focus Groups

Focus groups were used to explore the perspectives of parents and educators on the school participation of primary school students on the autism spectrum and to seek recommendations regarding the content and delivery of the intervention (Hodges et al., 2020). Four separate focus groups involving a total of 26 participants were conducted in Perth, Western Australia. Two focus groups were conducted with a total of 15 parents of children on the autism spectrum attending mainstream primary school. Two focus groups were conducted with a total of 11 educators including teachers (n = 5), deputy principals (n = 1) and learning support coordinators (n = 5) who reported having experience working with primary school students on the spectrum in a mainstream setting.

Parents and educators identified several intrinsic (e.g., students school connectedness and sense of self) and extrinsic (e.g., school culture and educator attitudes, knowledge, and skills) factors impacting the school participation of primary school students on the autism spectrum and emphasised the importance of developing school-based interventions that focus on addressing the psychological aspects of students’ school experience (Hodges et al., 2020). More detailed findings are reported elsewhere (Hodges et al., 2020) and helped to verify and enrich school participation barriers identified from the literature in the MSPA.

Parents and educators also provided general recommendations, which informed the overall approach of the intervention as well as the content, dosage (i.e., frequency and intensity) and method of delivery of the professional learning and whole class components of the intervention. Recommendations regarding ways to increase uptake of the intervention from parents’ and educators’ perspectives were also provided. Overwhelmingly, parents and educators felt the intervention should adopt a strengths- and differences-based approach, focusing on raising students’ awareness, understanding and acceptance of autism. Educators emphasised the importance of embedding lesson content into the curriculum with specific reference to curriculum outcomes in the manual and providing ideas on ways to individualise lesson content to the diverse needs of students and classrooms. To maximise uptake of the intervention, educators suggested resources need to be ‘ready to go’ with comprehensive lesson plans and printable resources to minimise burden for teachers (Hodges et al., 2020). This information was used to develop a more detailed description of the intervention, including: (a) a revised list of whole class lesson topics, (b) proposed content of professional learning, (c) weekly parent information handouts, and (d) proposed method of delivery of intervention components. These findings helped to guide avenues of questioning in the next phase of the research, which involved a national Delphi study.

### Delphi Study

Consensus from expert clinicians, researchers and educators was obtained on the content, delivery and feasibility of the intervention using an online two-round national Delphi study. Round one (clinicians, n = 34; researchers, n = 17; educators, n = 25; total experts, n = 76) focused on seeking expert opinion on the application of the fPRC to students on the autism spectrum. This round also provided evidence to support the relevance of the intervention, with all experts agreeing that improving the school participation of students on the autism spectrum, is important enough to warrant the development of an intervention and that school connectedness is not currently addressed in Australian curriculum. Round two (clinicians, n = 27; researchers, n = 18; educators, n = 20; total experts, n = 65; response rate = 87%) focused on gaining expert opinion on the importance of proposed whole class lesson topics and the feasibility of implementing proposed intervention techniques. More than 90% of experts agreed with the proposed content for lesson topics and reported intervention techniques were feasible or very feasible in the school environment. More detailed findings from the Delphi study are reported elsewhere (Hodges et al., 2021) and helped to develop and refine intervention components. For example, the Delphi study helped to determine that whole class lesson topics would be delivered in short (i.e., less than 60 min) regular sessions over the course of a term and that professional learning would focus on helping teachers to apply intervention content to their classroom and discuss ways the intervention can be practically incorporated into a school day.

### Feedback from Students, Parents, and Educators on Intervention Resources

Feedback on intervention resources was obtained from students, parents, and educators (i.e., teachers, deputy principals, learning support coordinators) so that the intervention could be refined prior to a feasibility study. Educators’ perspectives were also obtained on proposed data collection methods for the feasibility study.

Worksheets from the whole class component of the intervention were trialled with five typically developing primary school students for clarity of instruction and comprehensibility. These students were recruited using convenience sampling through networks of the primary author. Minor alterations were made to wording and formatting of the worksheets based on students’ feedback. Authors planned to seek feedback on the intervention from students on the autism spectrum, via online surveys and qualitative interviews, once the intervention had been piloted in primary schools. After having first-hand experience with the intervention, students would be able to reflect on their own experiences and provide feedback on how the intervention could be improved; avoiding hypothetical questions, which many students on the autism spectrum find difficult. Future iterations of the intervention will incorporate feedback from students on the autism spectrum to refine the intervention and improve outcomes in future research.

Weekly parent information handouts and the intervention manual were reviewed by parents and educators respectively using online surveys. Parents and educators were recruited using convenience and snowball sampling through networks of the primary author. Recruited parents and educators were also asked to identify other potential parents and educators. Potential participants were sent an email with an invitation to participate. Once they consented, the primary author sent through relevant intervention resources with a personalised link to an online survey (Qualtrics XM, 2021). The survey asked participants to respond to statements about the intervention resources on a 5-point Likert scale (1 = strongly agree to 5 = strongly disagree). For example, educators were asked to respond to statements such as “The manual was easy to read”, “I understood content of lesson plans”, and “I understood the examples provided in the professional learning and how these examples linked to the content”. Participants were prompted to provide reasoning for their responses if they selected ‘neither agree nor disagree’, ‘somewhat disagree’ or ‘strongly disagree’.

A combination of quantitative and qualitative approaches was used to analyse survey responses. Survey responses were imported into the Statistical Package for the Social Sciences (SPSS) (IBM Corporation, 2015) software and anonymised prior to analysis. Descriptive statistics were used to report participants’ responses to Likert scale items and agreement was reached (i.e., no changes were made to intervention resources) when more than 75% of participants responded ‘strongly agree’ or ‘somewhat agree’ to survey items. Content analysis was used to analyse participants written responses to identify recommended changes to specific intervention resources.

Eleven parents and 10 educators provided feedback on the intervention. Seven parents had children in years 1 to 3 and three of the 11 parents had a child with a diagnosed disability. Five educators were teachers from independent schools and six of the 10 educators had more than 10 years’ experience in their current role.

Parent feedback on weekly information handouts and proposed parent engagement was positive and agreement was reached on all survey items (see SI Table 1). More than 90% of parents reported parent information handouts were easy to read, that information was relevant and that they understood the content as well as examples provided and how these linked to the content. More than 80% parents reported they felt they could apply strategies at home with their children and that proposed methods of parent engagement were appropriate. Two parents raised concerns in qualitative comments over the depth of information provided, suggesting researchers condense and chunk information so that it is more visually appealing for parents.

Educators provided valuable feedback on the intervention manual, lesson plans, professional learning, and resources and agreement was reached on all survey items (see SI Table 2). All educators reported intervention resources were easy to read, engaging, that they understood the content and examples provided and that the type and depth of information were appropriate. Two of the 10 educators expressed concern that time allocated to lesson plans was unrealistic and reported time management would depend on teachers’ skills and experience. Educators reported, however, that lesson plans were thorough and allowed for flexibility and that teachers were able to use their judgement to modify or extend students. All educators reported understanding the proposed methods of data collection for the feasibility study, however, expressed concern about the amount of time it would take to administer measures with the whole class. We used these findings to make changes to the intervention manual, such as emphasising key messages of each lesson, highlighting mandatory activities and opportunities for individualisation. We also reviewed data collection methods for the feasibility study and reduced the number of whole class measures to minimise burden for teachers.

## The Resulting Intervention: *In My Shoes*

Based on the above research activities, the school-based intervention, entitled *In My Shoes,* has been developed (Hodges et al., 2020). *In My Shoes* aims to improve the school participation of primary school students aged between 8 and 10 years (grades 3 and 4) on the autism spectrum and their typically developing peers. The intended outcomes of *In My Shoes* for *all* students are to:

- increase understanding and awareness of differences in the way students experience autism and school *(i.e., preferences)*
- increase feelings of being accepted, respected, included and supported by others in the school social environment *(i.e., school connectedness);*
- increase self-awareness of strengths and differences and the strengths and differences of peers *(i.e., sense of self);*
- improve confidence in their abilities to recognise when someone needs help, how to help others and ask for help at school *(i.e., sense of self and activity competence)*; and
- improve students’ interpersonal empathy and use of pro-social behaviours to include peers in the classroom and playground *(i.e., activity competence).*

Intervention outcomes are specifically linked to intrinsic student factors impacting school participation outlined in the MSPA (see Fig. 1).

*In My Shoes* is designed to be delivered over the course of a school term (approximately 10 weeks) and includes the following components: (1) standardised online professional learning and ongoing face to face or online support for teachers and school leadership staff; (2) teacher-led whole class lesson plans; (3) peer training for selected peers; (4) activity ideas to incorporate key messages across the whole school; and (5) weekly parent information handouts and invitations for parents to participate in the intervention. Intervention resources are made available to schools on a USB memory stick and include professional learning video presentations, an online interactive PDF manual, printable lesson plans, worksheets and resources, and interactive video resources with real-life students on the autism spectrum sharing their school experiences.

*The professional learning component* encompasses all intervention outcomes, aiming to support teachers’ understanding of the content of *In My Shoes* and increase their capacity to utilise intervention techniques to support the school participation of students on the autism spectrum. The professional learning component includes supplementary pre-reading material detailing school participation barriers that result from characteristics of autism and evidence-based intervention techniques to support students on the autism spectrum in the classroom. Additionally, the resources include four pre-recorded video presentations (ranging from 4 to 24 min) of the primary author explaining the intervention and providing practical demonstrations of intervention techniques such as video modelling. Teachers are encouraged to complete a pre-post professional learning questionnaire that evaluates their adherence to reviewing supplementary material and the intervention manual, as well as their confidence in delivering specific components of the intervention. The purpose of these questionnaires is to identify teachers’ perceived barriers to implement the intervention so that the primary author can provide targeted support to teachers. School leadership staff involved in supporting teachers to deliver the intervention (e.g., deputy school principals, school psychologists or learning support coordinators) are also encouraged to complete the professional learning so that they can adequately support teachers and assist in implementing the whole school component of the intervention. The primary author then organises follow up online or face-to-face meetings with teachers and school leadership staff to clarify any components of the intervention and to help teachers apply concepts in their classroom.

**Fig. 3:**
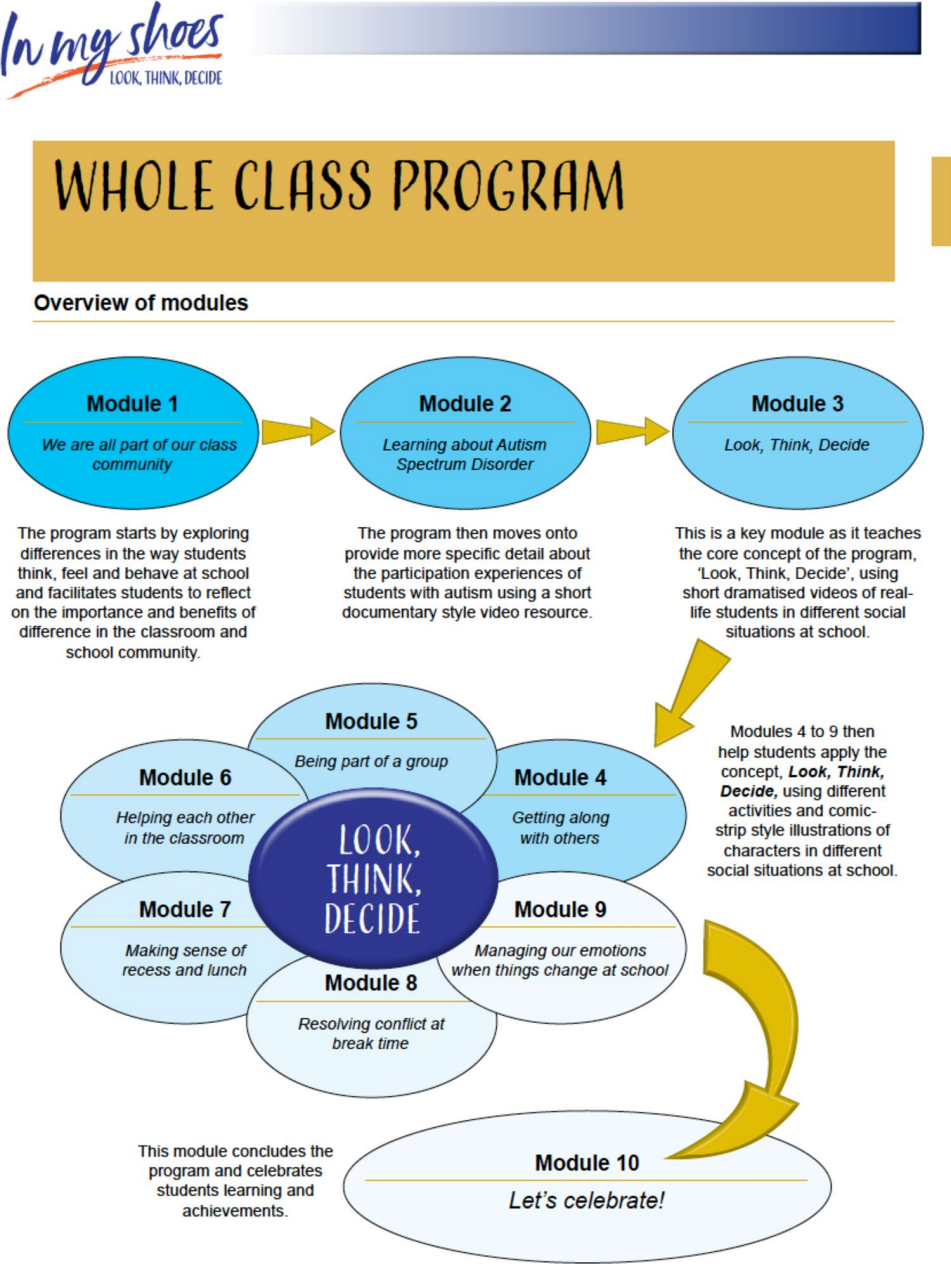
Overview of whole class lesson topics

*The whole class component* includes 10, 45-min lesson plans designed to be delivered by the classroom teacher to the whole class (see Fig. 3 for an overview of lesson topics). Each whole class lesson plan is designed to target *specific* intervention outcomes. Some lesson plans focus on targeting one intervention outcome, whereas others target several intervention outcomes. Over the 10 lesson plans, all intervention outcomes are targeted several times using a range of evidence-based intervention techniques including role play and video modelling, as well as educational practices identified to be feasible by educators (e.g., worksheets, whole class discussion). The whole class component starts by helping students to increase self-awareness of their strengths and differences and that of their peers *(i.e., sense of self);* focusing on celebrating student differences; reflecting on how each student adds value to the classroom, and identifying behaviours that make peers feel included, accepted, and valued for their differences *(i.e., school connectedness*). Students then learn about autism and how students on the autism spectrum experience school, hearing real-life students’ perspectives on a documentary style video. Lessons then progress to teaching the core concept of the intervention, ‘look, think, decide’, which teaches perspective taking and social problem-solving skills by helping students to recognise body clues and how to use these clues to deduce what someone else might be thinking and feeling so that they can decide on the best course of action to help peers participate and feel included. Students are asked throughout the intervention to reflect, using interactive video resources and comic-strip style illustrations, on what they would think or how they would feel if they were in a particular character’s shoes and what they think the character should do to support their peers in different situations. Each lesson aims to teach these skills with a particular context in mind; for example, how to recognise and support peers in the classroom versus the playground versus school organised events such as excursions, assemblies, or sports carnivals. Finally, lesson plans highlight opportunities to incorporate students’ preferences by building connections with peers who have similar interests and encouraging teachers to incorporate students’ strengths and interests into activities wherever possible.

The content of whole class lesson plans align with social emotional learning principals, which are an integral part of education and human development (Jones & Bouffard, 2012; Pasi, 2001); supporting students to acquire and apply knowledge, skills and attitudes to develop healthy identities, manage their emotions, feel and show empathy for others and establish and maintain supportive relationships at school. Links to state and national curriculum and social emotional learning competencies are explicitly referred to at the beginning of each lesson plan for teachers’ assessment and reporting requirements. Teachers are also provided with examples in the intervention manual on ways they can adapt or individualise lesson plans, in line with principals of universal design (Center for Applied Special Technology, 1998; Orkwis, 2003), to meet the diverse learning needs of students in their classroom. Refer to SI Table 3 for an example of a whole class lesson plan, detailing target intervention outcomes, specific objectives, and methods of delivery.

*The peer training component* of the intervention focuses on supporting selected peers to further build on their interpersonal empathy and use of pro-social behaviours *(i.e., activity competence)* to support students in the classroom and playground. This component of the intervention includes information about the benefits of peer involvement in school-based interventions and guides the teacher to carefully select three to four students in their class who consistently attend school, have a history of being reliable and responsible, may be interested and willing to help peers, have strong social and interpersonal skills, and have similar interests to target students. Selected peers participated in a teacher-led short informal discussion-based training in the first week of the intervention. The content of the training focuses on helping students to identify when someone looks lonely in the playground or are having difficulty in the classroom, and what they could do to help in these situations. The training draws on students’ previous experience and helps to highlight ways they may be able to help their peers at school.

*The whole school component* of the intervention includes information about the importance of school involvement for intervention outcomes, as well as recommended activity ideas to incorporate key messages of the intervention across the school. Activity ideas include example themes for assembly items, inserts for school newsletters about key messages of the intervention, and recommended books and resources for a library space about autism and neurodiversity. The whole school component aims to target all intervention outcomes over the course of the intervention. For example, an assembly item about ways students can make peers feel more accepted, respected and included at school would target the *school connectedness* intervention outcome, whereas a library space about autism would target the *preferences* intervention outcome by aiming to increase students’ understanding and awareness of autism.

*The parent component* of the intervention encompasses all intervention outcomes aiming to support parents to increase their understanding of the content of *In My Shoes* and ways they can support generalisation of skills in the home environment. This component includes weekly information handouts sent by teachers to parents detailing lesson content and regular opportunities for teachers to invite parents to participate in intervention specific activities. Teachers are also encouraged to check-in regularly with parents about their understanding of parent information handouts and provide regular feedback about students’ learning via photos or videos on school portals.

## Implications for Research and Practice

The imperative to develop a school-based intervention to improve the school participation of students on the autism spectrum arose from growing literature on the long-term negative impact of reduced school participation on student outcomes (Furlong et al., 2003; Maddox & Prinz, 2003; Shochet et al., 2006). We designed *In My Shoes* based on our own theoretical model of school participation and autism and a series of research activities, which aimed to gain iterative feedback from students, parents, educators, clinicians, and researchers with expertise in the topic area. The MSPA was imperative in defining constructs of interest to be targeted in the intervention and ensured the intervention was rooted in theory and evidence. Each step in the research process offered valuable comments and revisions to shape the intervention.

To participate at school, students need to have necessary skills and abilities, have self-determination, positive self-esteem and feel confident and satisfied in their abilities at school, feel accepted, respected, included, and supported by teachers and peers, and have interests or activities that hold meaning to them (Hodges et al., 2020, 2021). Rather than focusing on school participation barriers or students’ skills in isolation (illustrated from left to centre in Fig. 1), *In My Shoes* utilises a strengths-based approach to holistically promote school participation enablers using evidence-based intervention techniques (illustrated from right to centre in Fig. 1). The deliberate decision to immerse *all* students, not just those on the autism spectrum, in learning that focuses on behaviour and knowledge change, was important in shifting perceptions that students’ school participation occurs in isolation. More accurately, that it is a collective effort of all individuals within the school environment to help others participate and feel included at school. Framing lesson content around the tasks, activities and routines in which students participate, rather than the skills they need to participate, shifts the focus away from individual performance components; thereby allowing us to adopt a more functional approach to support student school participation. In this way, we can focus on how individuals within the environment can support each other to learn new skills, build positive self-esteem and feelings of being accepted, respected, and included at school.

The involvement of consumers was crucial in developing and refining the intervention (Consumer & Community Health Research Network, 2017). Expert recommendations from the Delphi study (Hodges et al., 2021) and feedback on intervention resources from students, parents and teachers invaluable in providing practical suggestions to ensure the intervention would be relevant, appropriate, and meet the needs of end users. Although we received feedback from many stakeholders including students, one that could have been improved was that of students on the autism spectrum. We plan to seek feedback from students on the autism spectrum once the intervention is piloted in primary schools; this way, students can reflect on their own experiences and provide feedback to improve the intervention and the potential outcomes of future research. We also suggest future research aims to form a working party of students on the autism spectrum across year levels to provide feedback on the intervention and its resources. This would help to better understand students’ lived school experiences (Fletcher-Watson et al., 2018), the practicalities of how the intervention would be perceived by students and their peers and provide invaluable feedback on the intervention and its resources.

The next step of the research process is to evaluate the feasibility, fidelity, and preliminary effectiveness of *In My Shoes* in mainstream primary schools. Once a feasibility study is conducted, we will be able to evaluate the interaction between constructs and the relationships illustrated in the MSPA and revise the model accordingly. Despite increased emphasis on the use of evidence-based interventions in schools, there continues to be widespread implementation of interventions that lack a strong theoretical rationale or that have minimal evidence to support their effectiveness (Odom et al., 2010). The process we undertook to identify and define constructs of interest and mechanisms to effect change in these constructs was integral in ensuring intervention had a strong theoretical rationale; helping us to communicate how and why we think the intervention is likely to work (Campbell et al., 2007). The MSPA and intervention development process described in this paper, can be used by other researchers, clinicians, and educators as a guide to develop interventions to support the school participation of students on the autism spectrum.

## Conclusion

A novel curriculum embedded peer-supported school-based intervention, entitled *In My Shoes,* that aims to improve the school participation of students on the autism spectrum and their typically developing peers has been developed from this multi-stage iterative research process. A theoretical model illustrating the interactive process between characteristics of autism and factors that promote school participation is also presented. The impetus to develop interventions with a strong theoretical rationale and next steps for research are discussed.

## Supplementary Information

Below is the link to the electronic supplementary material.

Supplementary file1 (DOCX 23 kb)
