# Supplementary material for: Closing the Gap Between Theory and Practice: Conceptualisation of a School-Based Intervention to Improve the School Participation of Primary School Students on the Autism Spectrum and Their Typically Developing Peers
Source: J Autism Dev Disord. 2021 Dec 4;52(7):3230–45. doi: 10.1007/s10803-021-05362-5 (PMC9213293; doi:10.1007/s10803-021-05362-5)
Supplement: Supplementary file 1 — Supplementary file1 (DOCX 23 kb) [file 10803_2021_5362_MOESM1_ESM.docx]

SI Table 1 Summary of Online Parent Feedback on Parent Information Handouts

|  | **Response (%)** | | | | |
| --- | --- | --- | --- | --- | --- |
| **Questions**  **Parents (n=11)** | **SA** | **SWA** | **NAND** | **SWD** | **SD** |
| The parent information handouts are easy to read | 46 | 54 | 0 | 0 | 0 |
| The parent information handouts are presented in a way that is engaging | 62 | 30 | 0 | 8 | 0 |
| I understood the content of the parent information handouts | 85 | 15 | 0 | 0 | 0 |
| I understood the examples provided in the parent information handouts and how these examples linked to content | 78 | 22 | 0 | 0 | 0 |
| The type of information provided in the parent information handouts is relevant | 62 | 38 | 0 | 0 | 0 |
| The depth of information provided in the parent information handouts is appropriate | 62 | 22 | 8 | 0 | 8 |
| I would be able to apply at least some of the suggested strategies on the parent information handouts to help generalise my child’s learning from In My Shoes to the home environment | 69 | 8 | 8 | 0 | 0 |
| The proposed methods of parent involvement in In My Shoes are suitable | 62 | 15 | 8 | 0 | 0 |
| *Note. SA= strongly agree; SWA = somewhat agree; NAND = neither agree nor disagree; SWD = somewhat disagree; SD = strongly disagree* | | | | | |

SI Table 2 Summary of Online Educator Feedback on Intervention Manual and Data Collection Procedures

|  | **Response (%)** | | | | | |
| --- | --- | --- | --- | --- | --- | --- |
| **Questions**  **Educators (n=10)** | **SA** | **SWA** | **NAND** | | **SWD** | **SD** |
| ***Intervention manual*** | | | | | | |
| The manual is easy to read | 80 | 20 | 0 | | 0 | 0 |
| The manual is easy to navigate | 60 | 20 | 20 | | 20 | 20 |
| The manual is presented in a way that is engaging | 70 | 30 | 0 | | 0 | 0 |
| The type of information provided in the manual is relevant | 70 | 30 | 0 | | 0 | 0 |
| The depth of information provided in the manual is appropriate | 90 | 10 | 0 | | 0 | 0 |
| I understood the content of the manual | 90 | 10 | 0 | | 0 | 0 |
| I understood the examples provided in the manual and how these examples linked to content | 90 | 10 | 0 | | 0 | 0 |
| I understood instructions in how to use the manual | 70 | 30 | 0 | | 0 | 0 |
| ***Lesson plans*** | | | | | | |
| The lesson plans are easy to read | 60 | 40 | 0 | | 0 | 0 |
| The lesson plans are presented in a way that is engaging as a teacher | 60 | 40 | 0 | | 0 | 0 |
| I understood the content of the lesson plans | 90 | 10 | 0 | | 0 | 0 |
| I understood the examples provided in the lesson plans and how these examples linked to the content | 90 | 10 | 0 | | 0 | 0 |
| I understood instructions in how to deliver the lesson plans to students | 90 | 10 | 0 | | 0 | 0 |
| The 45 minute time allocation for lesson plans is realistic | 30 | 50 | 10 | | 10 | 0 |
| The time allocated for individual activities in lesson plans is realistic | 50 | 30 | 10 | | 10 | 0 |
| The type of activities included in lesson plans are age appropriate | 70 | 30 | 0 | | 0 | 0 |
| The worksheets and resources are presented in a way that is fun and engaging for students | 50 | 50 | 0 | | 0 | 0 |
| The PowerPoint resource provided, to use as an additional visual support while teaching lessons, is useful | 80 | 20 | 0 | | 0 | 0 |
| There is sufficient detail in lesson plans about ways to scaffold students learning | 70 | 30 | 0 | | 0 | 0 |
| Links to state and national curriculum in lesson plans is clear and accurate | 90 | 10 | 0 | | 0 | 0 |
| *Supplementary pre-reading* | | | | | | |
| The supplementary information is easy to read | 60 | 40 | 0 | | 0 | 0 |
| The supplementary information is presented in a way that is engaging | 70 | 20 | 10 | | 0 | 0 |
| I understood the content of the supplementary information | 90 | 10 | 0 | | 0 | 0 |
| The type of supplementary information provided is relevant | 90 | 10 | 0 | | 0 | 0 |
| The depth of supplementary information provided is appropriate | 80 | 20 | 0 | | 0 | 0 |
| The type of information provided in online professional earning presentations is relevant | 90 | 10 | 0 | | 0 | 0 |
| The depth of information provided in online professional learning is appropriate | 90 | 10 | 0 | | 0 | 0 |
| I understood the content of online professional learning presentations | 90 | 10 | 0 | | 0 | 0 |
| I understood the examples provided in the professional learning and how these examples linked to the content | 90 | 10 | 0 | | 0 | 0 |
| I understood instructions in how to complete online professional learning presentations | 70 | 30 | 0 | | 0 | 0 |
| The professional learning is presented in a way that is engaging | 80 | 20 | 0 | | 0 | 0 |
| Please indicate how you would prefer to access In My Shoes professional learning in the future | All online (20) | | As provided (80) | | | |
| Please indicate how you would prefer to access In My Shoes manual and resources if you were implementing the program in your classroom | Electronic/ soft copy (20) | | | Both (80) | | |
| Please indicate your preferred use of language to refer to students/ with autism in the In My Shoes manual (select as many that apply) | Identity first (10%) | | | Person first (90) | | |
| Is there any information that you expected to see in the supplementary information that you did not? | No (100) | | | | | |
| Is there any information you expected to see in the online professional learning presentations that you did not? | No (100) | | | | | |
| Is there any content you expected in the manual that is not? | No (100) | | | | | |
| *Note. SA= strongly agree; SWA = somewhat agree; NAND = neither agree nor disagree; SWD = somewhat disagree; SD = strongly disagree* | | | | | | |

**SI Table 3 Example whole-class lesson plans including intervention outcomes, specific objectives and method of delivery**

|  | **Intervention outcomes** | **Specific objectives** | **Method of delivery** |
| --- | --- | --- | --- |
| **Module 3**  *Being part of a group* | - increase self-awareness of strengths and differences and the strengths and differences of peers *(i.e., sense of self);* - improve students’ interpersonal empathy and use of pro-social behaviours to include peers in the classroom and playground *(i.e., activity competence)* | At the end of this module students will:   - Understand some people have more difficulty than others understanding rules, understanding other people’s points of view and being flexible which can cause conflict in groups - Take the perspective of others who find group work difficult or do not enjoy group work - Identify warning signs that group work in class is not going well - Identify and practise strategies or ways of resolving conflict in groups and - Reflect on the importance and benefit of working together and being part of a group at school | - Students participate in a group activity where they have to work together to create a structure out of provided materials. - ***Role play –*** one group member (without his/her peers knowledge) is required to act like a character from *In My* Shoes with the aim of causing conflict in the group. - ***Video modelling –*** the classroom teacher videos one group participating in the activity and plays back the video to students at the help. The teacher pauses the video at key points to help students pinpoint specific body clues (e.g., facial expressions, body language, tone of voice, volume or voice, actions or behaviour) of students that help them know the group work is starting to break down. - ***Whole class discussion –*** students brainstorm strategies that students could have used to manage conflicts or difficulties and the good things that could have happened if they chose these actions. |
